# Dynamic analysis of immune status in patients with intracranial germ cell tumor and establishment of an immune risk prognostic model

**DOI:** 10.3389/fimmu.2022.1010146

**Published:** 2022-10-11

**Authors:** Hairong Wang, He Huang, Xiaoping Lin, Peidong Chi, Hongyu Chen, Jiangen Chen, Yonggao Mou, Zhongping Chen, Qunying Yang, Chengcheng Guo

**Affiliations:** ^1^ Department of Neurosurgery/Neuro-oncology, State Key Laboratory of Oncology in South China, Collaborative Innovation Center for Cancer Medicine, Sun Yat-sen University Cancer Center, Guangzhou, China; ^2^ Department of Medical Oncology, State Key Laboratory of Oncology in South China, Collaborative Innovation Center for Cancer Medicine, Sun Yat-sen University Cancer Center, Guangzhou, China; ^3^ Department of Nuclear Medicine, State Key Laboratory of Oncology in South China, Collaborative Innovation Center for Cancer Medicine, Sun Yat-sen University Cancer Center, Guangzhou, China; ^4^ Department of Clinical Laboratory, State Key Laboratory of Oncology in South China, Collaborative Innovation Center for Cancer Medicine, Sun Yat-sen University Cancer Center, Guangzhou, China

**Keywords:** intracranial germ cell tumors, lymphocyte subsets, prognosis, adolescent tumors, dynamic changes

## Abstract

**Introduction:**

Immune status was evaluated by means of lymphocyte subset counts and immune factors in cancer. This study analyzed the peripheral blood immune index and survival outcomes in intracranial germ cell tumor (iGCT) patients.

**Methods:**

Peripheral blood lymphocyte subset counts and levels of interleukin (IL)-2, IL-4, IL-6, IL-10, tumor necrosis factor (TNF), and interferon-γ (IFN) from 133 iGCT patients were collected and retrospectively analyzed. Their clinical information was extracted from the hospital database, and prognosis was confirmed by telephone visit. Patients (n=11) underwent prospective review and their samples of peripheral blood lymphocytes were verified.

**Results:**

A total of 113 (84.2%) patients received comprehensive treatments, including 96 standard therapy (combination of full course chemotherapy and radiology with or without surgery) and 17 comprehensive but non-standard therapy (either without full course chemotherapy or with non-standard radiotherapy) and 98 (73.7%) reached complete or partial response. T lymphocytes (CD3^+^), cytotoxic T cells (CD3^+^CD8^+^ or Tc), and B lymphocytes (CD19^+^) decreased (p=0.047, p=0.004, and p<0.001, respectively), while activated cytotoxic T lymphocytes (CD8^+^CD25^+^) and IFN increased (p<0.001 and p=0.002, respectively) after treatment. Median survival was 45.33 months, and patients with increased Tc cells and activated Tc cells as well as IFN presented encouraging outcomes (p=0.039, p=0.041, and p=0.017 respectively). Regression analysis showed that non-increased Tc cells and non-increased activated Tc cells were independent factors of poor prognosis (p=0.016, HR=3.96, 95%CI=1.288-12.20; p=0.002, HR=4.37 95%CI= 1.738-10.97). Standard chemo-radiotherapy was independently related to reduced risk of death(p=0.022, HR=0.19, 95%CI=0.044-0.79). Consistence was seen in a nomogram established through retro and prospective studies. An immune risk model indicated the activated group (with both increased activated T cells and IFN levels) had the best prognosis, the mildly activated type with elevated IFN levels had intermediate outcome, and patients with the silent immune status had the worst outcomes (Log rank test, p=0.011).

**Conclusion:**

Implementation of standard comprehensive treatments led to positive responses. Dynamic monitoring of peripheral blood lymphocyte subsets can be used as an auxiliary indicator for prognosis judgment.

## Introduction

Intracranial germ cell tumors (iGCTs) are rare brain tumors that originate from embryonic germ cells. They are mainly seen in children and adolescents, with the highest incidence rate between 10-14 years old (1). The sellar region, pineal area, and the basal ganglia area are the most commonly affected areas, especially the former two (2–4). The incidence rate varies greatly between continents: iGCT is more common in Asia than in North America and Europe (5). The total incidence rate in the United States is 0.6 per million per year, Europe is 1.0 per million per year, and Japan is 2.7 per million per year (6). Classification of central nervous system germ cell tumors according to the World Health Organization divides them into germinoma and non-germinoma germ cell tumors (NGGCTs), with six different types of the latter, including teratoma, embryonal carcinoma, endodermal sinus tumor (yolk sac tumor), chorionic epithelioma (also called choriocarcinoma), and mixed germ cell tumors (mix GCTs) (7). Diagnostic methods vary by region; some countries rely on surgical (a gross total resection or biopsy) and pathological verification. With consideration of safety and prognosis, other countries look at tumor markers: α-fetoprotein (or AFP), which is typically raised in yolk sac tumors; and human chorionic gonadotropin (HCG), which is typically raised in the presence of choriocarcinomas. The tumor markers, together with steady radiological appearances and clinical characteristics, are used as confirmation of diagnosis. Commonly, increased HCG and/or AFP are viewed to be positively correlated with poor prognosis (8). However, it is unlikely that using simple tumor markers to evaluate patients’ changing conditions can predict outcomes precisely, not to mention the evaluation and prognostic significance of patients with negative tumor indicators. Moreover, though optimum management for iGCTs patients is recognized to depend on a collaborative team of experts from multi-disciplines, variable patterns increase the difficulty of personalized treatment decisions.

Lymphocyte subset measurements are commonly used in the evaluation of human immunodeficiency virus (HIV) infection (9), primary immunodeficiency diseases (10), autoimmune diseases (11), adenocarcinoma (12), and leukemia (13). In recent decades, it has been recognized that malignant tumor cells potentially induce local and systemic immunosuppression (14). Therefore, the application of immunotherapy has driven the treatment of brain tumors to the study of the response of intratumor and systemic immune cells and cytokines to these malignancies (15–17). Studies confirmed that many activated CD4^+^CD25^+^ T cells play a crucial role in anti-tumor immune responses and are of beneficial prognostic influence in non-small cell lung carcinoma (NSCLC) patients (18). However, increased CD4^+^CD25^+^ Treg cells related to augmentation of malignant cells in the tumor microenvironment and the presence of elevated CD25^+^ cells in peripheral blood are associated with chemoresistance in lung cancer (19), advanced renal carcinoma (20), breast cancer (21), gastrointestinal malignancies (22), and high virus load. Patients with higher intertumoral or circulating Tc cells generally have a slower progression and live longer (23). Accordingly, we speculate that an examination of the lymphocyte subset dynamics has practical meaning for monitoring the progression and prognosis of intracranial germ cell tumors and for helping with optimal clinical decision-making as an auxiliary indicator. The immunomodulated status of intracranial germ cell tumors is unclear, and further studies are needed to understand the relationship of the tumor immune subset and the peripheral blood immune subset as well as their survival outcomes.

## Materials and methods

### Patients

This was an observational study. Approval was granted by the Ethics Committee of Sun Yat-sen University Cancer Center (B2022-204-01). A total of 133 patients from the retrospective study and 11 patients from the prospective study (NCT04909307) diagnosed with intracranial germ cell tumors were included. Informed consent was obtained from all individual participants and parents (if patients were children) included in the study. They received comprehensive treatments at Sun Yat-sen University Cancer Center from June 2011 to November 2019. Patients with any of the following criteria were excluded: patients with immunosuppression status, including autoimmune disease, post-operation of organ transplantation, and intaking an immunosuppressive drug. Informed consent was obtained from all individual participants included in the study.

### Diagnoses, clinical study design, and treatment

**Figure 1:**
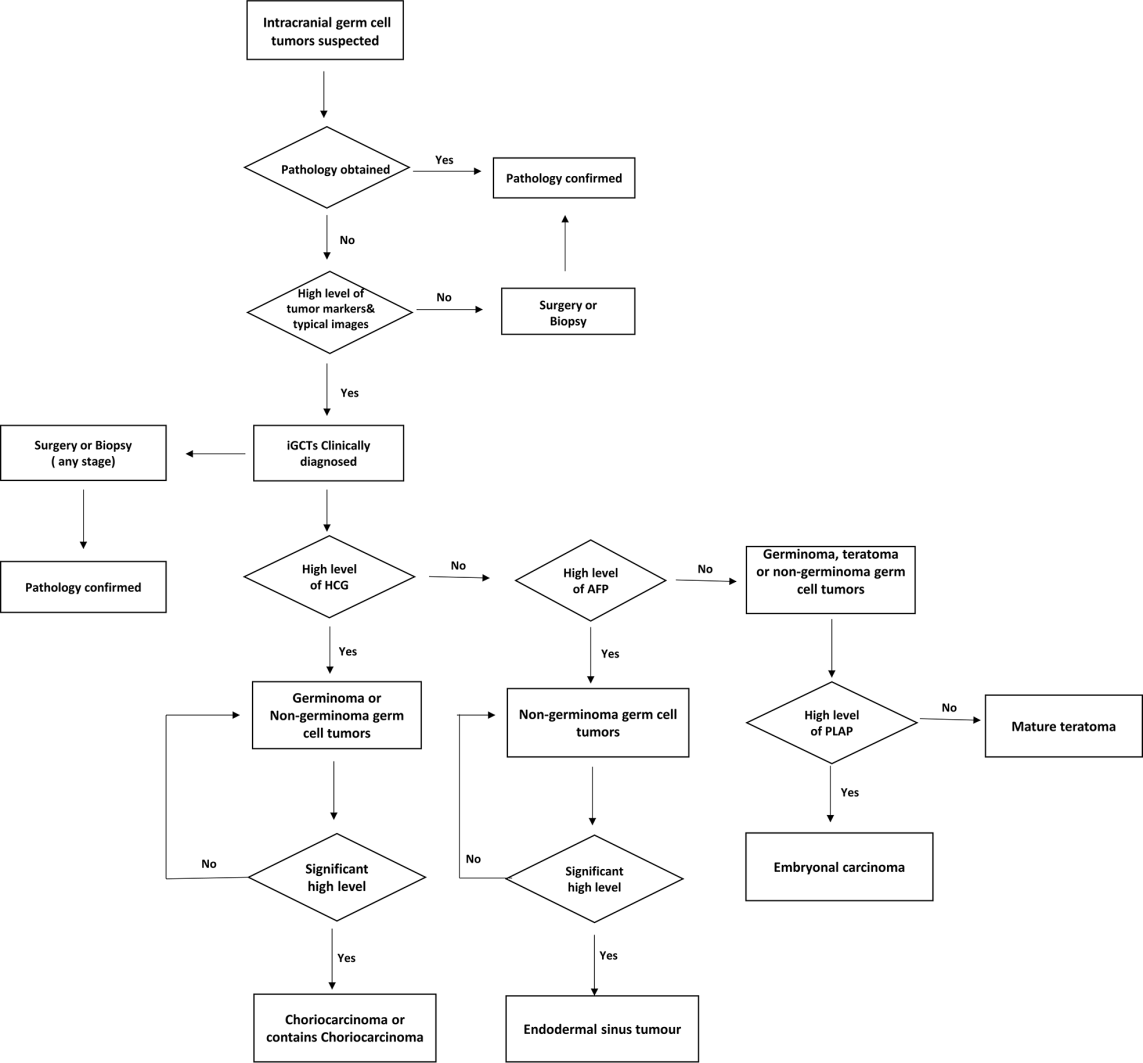
Diagnose flow chart of suspected intracranial germ cell tumor patients in Sun Yat-sen University Cancer Center.

**Figure 2:**
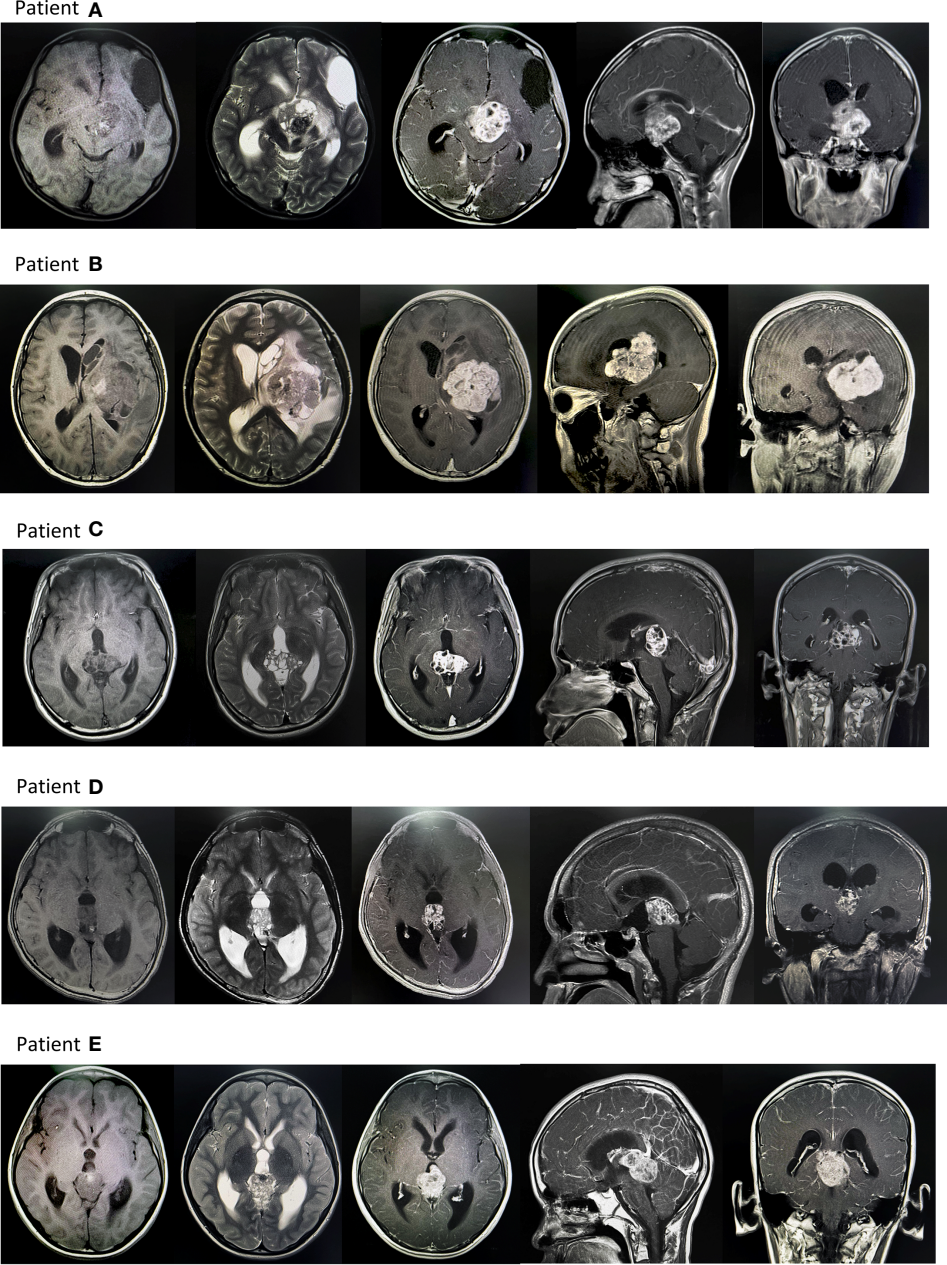
Typical MR images of intracranial germ cell tumors patients. Patient **(A)** There was an irregular cystic solid tumor in the suprasellar area, with a size of about 40mmx35mmx34mm, with clear borders, high and low mixed signals on T1WI, and high and low mixed signals on T2WI. After enhanced enhancement, the possibility of a germ cell tumor was considered. There are fusiform nodules in the left ventricle and the fourth ventricle, with clear borders, about 12mmx8mmx7mm, 9mmx8mmx16mm in size, and the enhancement is obviously uneven, and implantation is considered. Patient **(B)** Huge mass in the left basal ganglia with poorly defined borders, the largest slice is about 53mmx69mm: TIWI with uneven low signal, see strip-like high signal, T2WI with uneven high signal, see patch cystic lesions, uneven after enhanced scan Significant enhancement. Edema zone was seen in the brain parenchyma around the tumor. The possibility of germ cell tumor was considered. The left lateral ventricle was compressed and narrowed, and the midline structure was shifted to the right. Patient **(C)** An irregular mass was seen in the pineal region, with a size of about 24mmx35mmx32mm, with a clear boundary and uneven signal. T1WI showed equal and slightly low signal, and T2WI showed slightly high signal. The solid component was significantly enhanced on enhanced scan. The third ventricle is dilated and effusion, thus it is likely to be a reproductive tumor. Patient **(D)** Irregular soft tissue signal mass can be seen in the pineal region, with a clear boundary and a range of about 21mmx27mmx20mm. T1WI shows a slightly low signal, a few dots and a slightly high signal inside, T2WI shows a slightly high signal, and it shows uneven enhancement after enhancement, and germ cell tumor is considered. tumor. Obstructive hydrocephalus of the third ventricle and bilateral lateral ventricles. Patient **(E)** An irregular mass foci was seen in the pineal region, about 32mmx30mmx36mm in size. TIWI showed a low signal, and T2WI showed a mixed abnormal signal area of equal height, in which the liquid level was seen, the enhancement was obvious, and the cystic area was seen, and a germ cell tumor was considered. Occupying the great cerebral venous cistern, pushing the quadrigeminal cistern, and protruding into the third ventricle, compressing the midbrain aqueduct, causing double ventricles, dilation of the third ventricle, and obstructive hydrocephalus.

**Figure 3:**
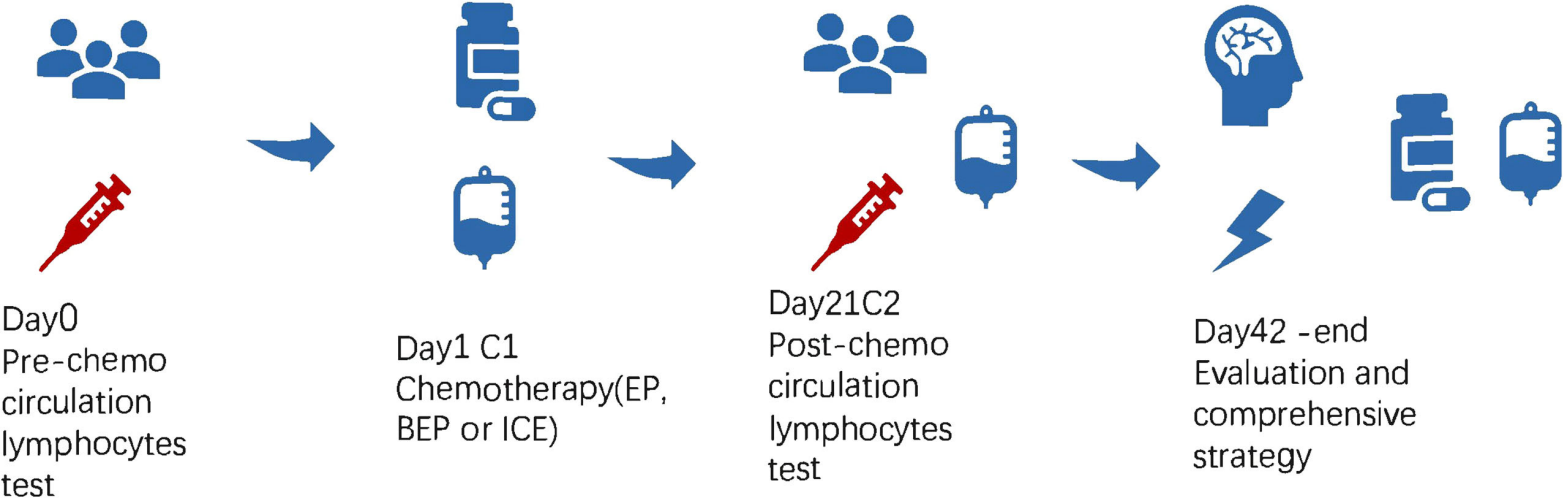
Whole process management method and experimental design.

In the retrospective study of 133 patients, we followed the flow chart presented in **Figure 1** to diagnose suspected iGCT patients. For cases that had not been confirmed by histopathology, we combined clinical symptoms (diabetes insipidus, polydipsia and polyuria, visual impairment, headache, vomiting, papilledema in male children), site of disease (pineal gland, saddle area, or basal ganglia), CT or MR images (focal mixed density or signal, uneven enhancement after enhanced scanning, cystic or central calcification, typical MR images shown in **Figure 2**) with peripheral blood or cerebrospinal fluid tumor indicators, comprehensively clinically diagnosed as germ cell tumors. The study design is shown in **Figure 3**: peripheral blood lymphocyte subsets and immune factors are tested before initial treatment and after 1 course of chemotherapy, and evaluated at the end of 2 courses of treatment. Comprehensive clinical management was customized for each patient as listed, until the development of unacceptable toxicity from therapies, withdrawal or death: a) platinum-based chemotherapy, etoposide, bleomycin, and cisplatin (carboplatin was recommended if patients suffered an adverse reaction to cisplatin), ifosfamide, and etoposide (EP, BEP, or ICE), alternating at 21-day intervals with cycles; b) surgery: tumor resections were primarily reserved for hypothesized iGCT patients with negative tumor markers, and ventricle-abdominal shunt was suggested for those who could not tolerate tumor resection; c) Radiotherapy was administered after 4-6 cycles of chemotherapy, followed by additional two cycles of anti-tumor drugs. Regarding target volume, either whole-brain radiotherapy (WBNT) 21.6-36Gy or craniospinal irradiation (CSI) 19.8-30.6 Gy with boost (16.2-23.4 Gy) were considered. The gross target volume (GTV) was defined as the extent of the primary tumor(s) before treatment. The clinical target volume (CTV) was obtained by adding 1–2 cm to the GTV. For patients who suffered from progression, we provided extra temozolomide (TMZ) and bevacizumab.

### Evaluation and follow up

An evaluation was performed every two chemotherapy cycles with the following: a) Peripheral blood lymphocyte subsets and tumor marker were compulsive and those in CSF were performed for patients without contraindication. b) Lymphocyte subsets were counted by flow cytometry analysis in center laboratory. A volume of 5 mL of ethylene diamine tetraacetic acid (EDTA) anticoagulated venous blood was collected within one day before the start of chemotherapy and the three weeks after treatment. c) Criteria for Response Assessment Incorporating Magnetic Resonance Imaging and Clinical Factors was used for evaluation. Overall survival (OS) was defined from the time of first anti-tumor treatment to death from any cause or last follow-up.

### Statistical analysis

IBM SPSS Statistics for Windows (version 25.0) and R (version 4.1.2) were used to perform the data analyses. Paired-sample t-test and one-way ANOVA, Wilcoxon test and Mann-Whitney U test, and X2 test were used. The Kaplan Meier method was used to calculate the OS curves, and the log-rank test was employed to assess differences. Cox regression was applied to multivariate analysis. A two-sided p-value < 0.05 was considered as significant.

## Results

### Patient characteristics and treatment details

A total of 133 patients were eligible for the retrospective study, and their characteristics are summarized in **Table 1**. Representative starting symptoms consisted of headache and vomiting (71/133, 53.4%), precocious puberty/abnormal menstruation in women/male libido (13/133, 9.8%), adipic diabetes insipidus (19/133, 14.3%), impaired vision (8/133,6.0%), hemiparesis (18/133, 13.5%), epilepsy seizure (4/133, 3.0%), and other atypical symptoms (chest tightness, memory loss, hiccup singulation, fever, etc.).

**Table 1:** General characteristics of iGCTs patients in retrospective study cohort.

General characteristics of iGCTs Patients									
Age	Min	Max	Median						
	5	44	16						
Gender	Male	110 (82.7%)							
	Female	23 (17.3%)							
Histology	GCT	68 (51.1%)				Stage	M0	85 (64.0%)	
	NGGCT	65 (48.9%)					M1	47 (35.3%)	
							Cannot be defined	1 (0.8%)	
Locations			Tc cells			p	Activated Tc cells		p
		Total	Increased		Non-increased	0.684	Increased	Non-increased	0.602
	Single at typical sites	70 (52.6%)	37 (27.8%)		33 (24.8%)		36 (27.1%)	34 (25.6)	
	Single tumor at atypical sites	7 (5.3%)	5 (3.8%)		2 (1.5%)		5 (3.8%)	2 (1.5%)	
	Violation of ventricle systems	25 (18.8%)	10 (7.5%)		15 (11.3%)		14 (10.5%)	11 (8.3%)	
	Bifocus	18 (13.5%)	6 (4.5%)		12 (9.0%)		12 (9.0%)	6 (4.5%)	
	Multiple tumors	13 (9.8%)	7 (5.3%)		6 (4.5%)		9 (6.8%)	4 (3.0%)	
Classification		Total	Increased		Non-increased		Increased	Non-increased	
	Germinoma	68 (51.1%)	32 (24.1%)		36 (27.1%)	0.319	41 (30.8%)	27 (20.3%)	0.655
	Teratoma	6 (4.5%)	2 (1.5%)		4 (3.0%)		4 (3.0%)	2 (1.5%)	
	Malignant teratoma	10 (7.5%)	6 (4.5%)		4 (3.0%)		4 (3.0%)	6 (4.5%)	
	Endodermal sinus tumor	6 (4.5%)	3 (2.3%)		3 (2.3%)		3 (2.2%)	3 (2.2%)	
	Embryonal carcinoma	3 (2.3%)	3 (2.3%)		0		1 (0.8%)	2 (1.5%)	
	Choriocarcinoma	8 (6.0%)	6 (4.5%)		2 (1.5%)		3 (2.3%)	5 (3.8%)	
	Mixed of germ cell elements	32 (24.1%)	13 (9.8%)		19 (14.3%)		20 (15.0%)	12 (9.0%)	
Tumor markers		CSF AFP elevation/CSF availability	11/108						
		CSF β-hCG elevation/CSF availability	57/108						
Status	Alive	103 (77.4%)							
	Death	30 (22.6%)							
Overall survival (months)	Min >0	Max 107

Typical sites: neurohypophysis, pineal region, and basal ganglia regions.

A total of 17 patients complained of recurrence of the disease, and eight of them had received surgery at the first visit. Time to progression ranged from 5 to 98 months, and the median time to progression was 27 months. Among them, one patient who had undergone surgery did not perform follow-up chemotherapy or radiation treatment, which led to progression within one year. In comparison, three patients completed the whole procedure of comprehensive therapy and lived over 60 months (to be exact, 67, 80, and 98 months, respectively). Neurohypophysis, pineal region, and basal ganglia regions were found as the three most prevalent areas5. Among the patients with recurrence of these diseases, the tumors of five patients were single lesions located in these common sites, and two patients’ tumors were located at uncommon sites (e.g., frontal lobe, parietal lobe). Only one patient whose tumors were multiple complained of recurrence.

The pattern of treatment details is summarized in **Table 2**. Treatment patterns notably associated with the evaluation of the disease (p=0.008*, **Supplementary Figure1A**). Two male patients had disease progression after standardized combination therapy, and despite the fact that a combination of surgery + chemoradiotherapy was taken, those patients who were not treated in a standardized way (inadequate course of treatment or irregular radiotherapy modalities, etc.) reduced the complete remission rate from 30.1% to 3.76%. Complete Remission cannot be achieved with surgery only or surgery + chemotherapy.

**Table 2:** The pattern of treatment details.

		No.	%
Therapy Pattern 1	Surgically removed+Chemotherapy+Radiotherapy	45	33.83%
	Ventricle-abdominal shunt+ Chemotherapy+Radiotherapy	15	11.28%
	Gamma knife+Chemothrapy+Radiotherapy	6	4.51%
	Chemotherapy+Radiotherapy	47	35.34%
	Surgically removed+Chemotherapy	11	8.27%
	Chemotherapy	9	6.77%
Therapy Pattern 2	Standard treatment	96	72.18%
	Comprehensive but non-standard treatment	17	12.78%
Chemotherapy	BEP	39	29.10%
	EP	62	46.62%
	Combination of BEP/EP/IEP/TMZ/Bevacizumab	15	11.28%
	IEP	2	1.50%
	NA	2	1.50%
Radiotherapy	CSI+boost	54	40.60%
	WBNT+boost	20	15.04%
	Local radiotherapy	5	3.76%
	No radiation	21	15.79%
	Not recorded	32	24.06%
At least 4-6 courses of Chemo	Yes	117	87.97%
	No	16	12.03%
Combination of Radiotherapy	Yes	112	84.21%
	No	21	15.79%
Relapse	Yes	17	12.78%
	No	116	87.22%
Outcomes of treatments	CR	47	35.34%
	PR	51	38.35%
	SD	28	21.05%
	PD	7	5.26%

### Dynamic changes in lymphocyte subsets and immune factors

**Figure 4:**
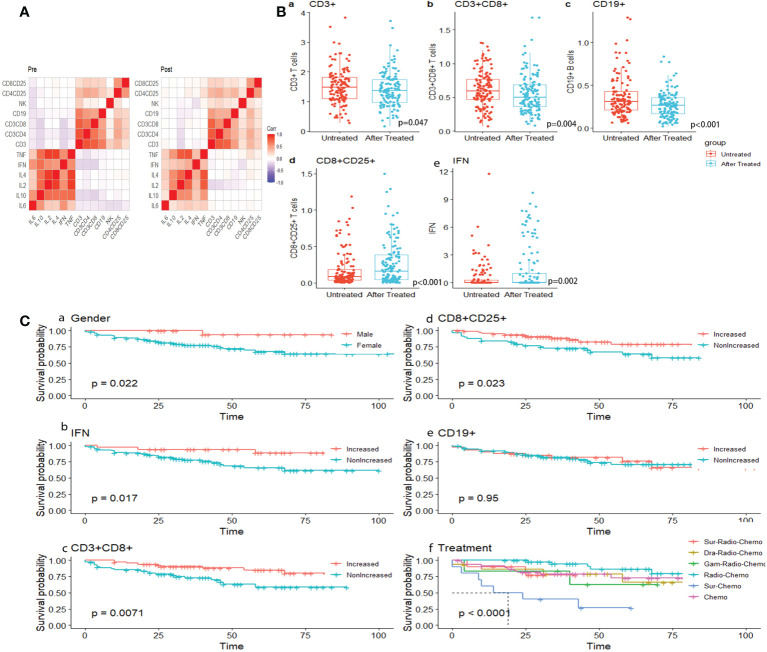
**(A)** Correlation heat map of immune indicators before and after treatment. **(B)** Dot plots showing dynamic surveillance of absolute lymphocyte subsets count in peripheral blood before and after one course of chemotherapy. **(C)** K-M survival analysis under gender, dynamic change of immune index and treatment.

Peripheral blood lymphocytes including T (CD3^+^) cells, cytotoxic T cells (Tc or CD3^+^CD8^+^T cells), and B (CD19^+^) cells significantly decreased (p=0.047, p=0.004, and p<0.001, respectively), compared with the augmentation of activated cytotoxic T cells (activated Tc or CD8^+^CD25^+^ T cells)(p<0.001) and IFN-γ (**Figure 4B**, **a–e**). Likewise, T helper cells (Th or CD3^+^CD4^+^ T cells), and natural killer cells (NK cells or CD3^-^CD16^+^CD56^+^ T cells), and IL6 and IL10 appeared to be reduced because of chemotherapy, although the change was not significant (p=0.97, p=0.121, p=0.437, p=0.446 (**Supplementary Figure 1B**). Activated T helper cells (activated Th cells or CD4^+^CD25^+^T cells), IL2, IL4, and TNF (tumor necrosis factor) increased (p=0.632, p=0.47, p=0.386, and p=0.957, respectively, **Supplementary Figure 1B**). These results indicate that inducing one course of chemotherapy to patients with germ cell tumors could restrain the proliferation of immune cells, especially T cells, while enhancing activated T cells. No differences were seen in lymphocytes subsets or immune factors between GCTs and NGGCTs either pre-/post- chemotherapy, except higher level of peripheral blood IFN in NGGCTs before chemotherapy(p=0.026 **Supplementary Figures 1C-F**). The prognosis of increased IFN-γ was significantly better in the NGGCT patients (p=0.0081). Forest plots created from cox multifactorial regression analysis suggested that non-increased IFN-γ and activated T cytotoxic T cells were at increased risk of death in NGGCT (IFN:p=0.05, HR=7.98. 95% CI=0.996-63.9; CD8+CD25+ T cells, p=0.032, HR=3.52, 95% CI=1.113-11.1)(**Supplementary Figure 2**)

### Survival analysis and nomogram

The median follow-up time was 45.33 months, ranging from 6 to 107 months (Log-rank test, p=0.022) (**Figure 4C**, **a**).

Dynamic alternation of lymphocytes indicated that patients with increased Tc cells, activated Tc cells, and increased peripheral blood level of IFN were inclined to present significantly encouraging survival outcomes (Log-rank test, p=0.0071, p=0.023, and p=0.017, respectively) (**Figure 4C**, **b–d**). The correlation between lymphocyte subsets and immune factors changed from negative to weak positive (**Figure 4A**). Similar results were seen in subsets of B cells (CD19+), though not significantly (p=0.96) (**Figure 4C**, **e**). The combination of radiotherapy and chemotherapy had the best survival results, which is statistically significant (p<0.001) (**Figure 4B**, **f**).

**Figure 5:**
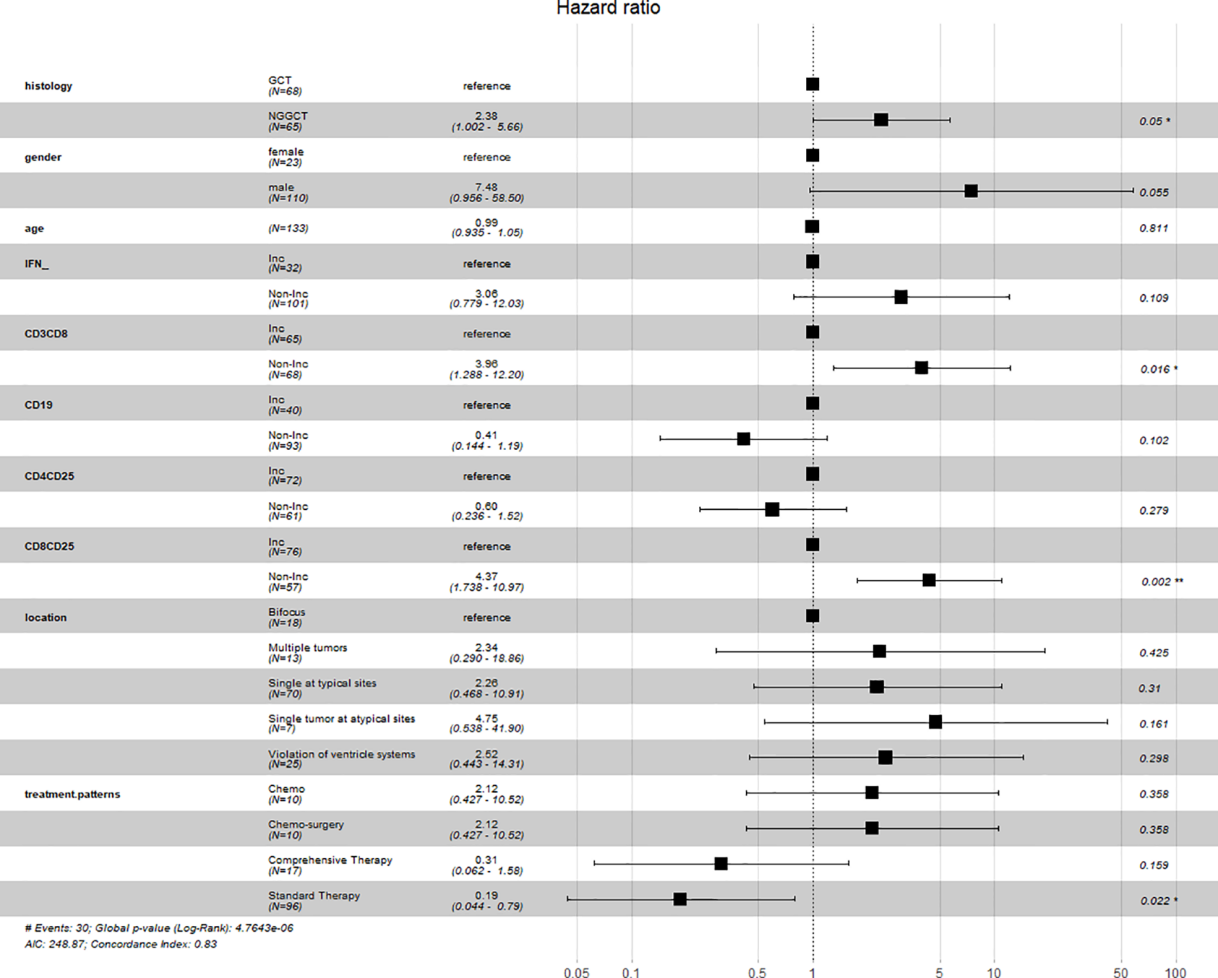
Forrest plot of multiple cox regression.

Considering that the changes in the level of IFN, Tc cells, B cells, and activated Tc cells were either statistically significant or associated with the survival outcomes of iGCT patients, multivariate Cox regression analysis was performed on these variables together with histology, tumor sites and choice of treatment as a panel to predict the prognosis (p<0.001) (**Figure 5**). Regression analysis showed that non-increased Tc cells and non-increased activated Tc cells were both considered as independent factors of poor prognosis (p=0.016, HR=3.96, 95%CI=1.288-12.20; p=0.002, HR=4.37 95%CI= 1.738-10.97). Though non-increased IFN levels were not seen as an independent prognosis factor, the levels indicated similar results of increased risk of death (p=0.109, HR=3.06, 95%CI= 0.799-12.03). Standard chemo-radiotherapy was independently related to reduced risk of death(p=0.022, HR=0.19, 95%CI=0.044-0.79).

**Figure 6:**
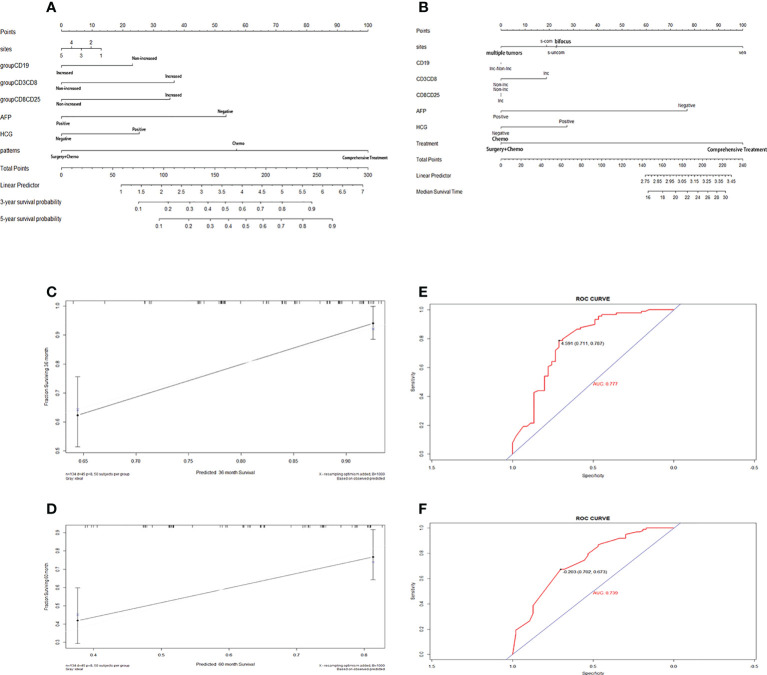
**(A)**. Nomogram predicting 3‐ and 5‐ survival for retrospective intracranial germ cell tumors patients. Codes for tumor location: 1 single tumor at pineal gland/basal ganglia/saddle; 2 Violation of ventricle systems; 3 Single tumor at others sites; 4 Bifocus tumor of pineal gland and saddle; 5 Multiple tumors; **(B)**. Nomogram predicting 3‐ and 5‐ survival for prospective intracranial germ cell tumors patients. **(C, D)**. The calibration curves for predicting patient survival at B(a) 3‐y and B(c) 5‐y. Nomogram‐predicted survival is plotted on the x‐axis; actual survival is plotted on the y‐axis. **(E, F)** ROC curves of nomogram panel and tumor makers(AFP plus HCG). New scoring system had higher accuracy compared with scoring system based on tumors markers.

To further visualize the prediction of the overall survival of iGCTs patients, a prognostic nomogram was established through the Cox regression model analysis according to those significant indicators above (Tc cells, B cells, activated Tc, peripheral blood AFP and HCG, tumor location and treatment patterns) (**Figure 6A**). Each factor in the nomogram was assigned a weighted number of points, and the sum of points for each patient was under a specific predicted 3- and 5-year OS. For internal validation, the bootstrapped calibration plot of the nomogram predicting 3- and 5-year OS performed well with the ideal model (**Figures 6C, D**). The C-index of the prognostic model was 0.781 ± 0.071, and the integrated AUC curves is shown in **Figure 6E**. In comparison, the C-index of tumor markers was 0.644 ± 0.081, and the integrated AUC curves are shown in **Figure 6F**. According to the nomogram model, lymphocyte-subset counts, tumor markers, and other factors from 11 patients included in the prospective study were applied to establish a new nomogram (**Figure 6B**) and calculate the scores and survival probability, while comparing them with their current actual status (**Table 3** and **Supplementary Table**).

**Table 3:** Nomogram score and survival probability calculation of dynamic peripheral blood lymphocyte changes in prospective patients, compared with actual survival status and survival time.

Nomogram Scores of Patients from Prospective Study				
	Scores	5-year Survival Possibility	Actual Status	Actual Survival Time/Follow-up Time
Patients1	257.5	85%	Survival	30
Patients2	242.5	82%	Survival	31
Patients3	312.5	>90%	Survival	28
Patients4	270	90%	Survival	29
Patients5	330	>90%	Survival	30
Patients6	192.5	55%	Survival	29
Patients7	265	87.5%	Survival	24
Patients8	307.5	>90%	Survival	24
Patients9	232.5	75%	Survival	24
Patients10	215	67.5%	Death	21
Patients11	115	15%	Death	16

**Figure 7:**
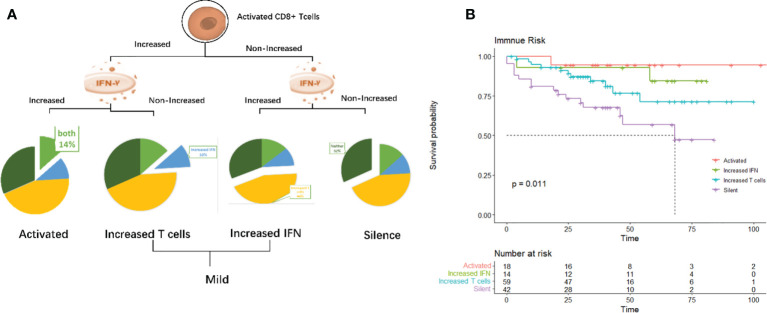
**(A)** Immuno-groups. group1: Activated (both activated T cells and IFN levels increased), group2 and group 3: Mild (group2: activated Tc cells increased; group3: IFN increased) and group 4:Silent (both activated T cells and IFN levels were not increased). **(B)**. K-M survival analysis of corresponding immuno-groups.

To describe more intuitively the significance of dynamic immune indicators in predicting the prognosis of iGCT patients, we established an immune risk model, that divided patients into four subgroups (**Figure 7A**). Group 1 included both increased activated T cells and increased IFN levels; Group 2 and Group 3 were considered mild (in group 2, activated Tc cells were increased; in group 3, IFN levels were increased); and Group 4 patients were considered silenced, in which neither activated T cells and IFN levels were increased. Survival analysis based on immune risk (**Figure 7B**) suggests that patients with immune activation had the best prognosis, patients with elevated IFN levels in the mild immune type had intermediate prognosis, and patients with the silent immune status had the worst outcomes.

## Discussion

Intracranial germ cell tumors (iGCT) are a rare heterogeneous group of neoplasms mostly located in the pineal and/or sellar regions and mainly occurring in children and adolescents (2, 24). Teilum established the so called “germ cell theory,” which posits that germ cell tumors emerged from primordial germ cells (PGCs) that mis-migrated to the midline of the body and led to CNS lesion occur (25). Similar global DNA hypomethylation was seen in both germinoma and in PGCs (26). Co-analysis with the transcriptome of human embryonic cells revealed that germinomas had expression profiles similar to those of primordial germ cells, while the expression profiles of NGGCTs were similar to those of embryonic stem cells (27). Mutations in the MAPK and PI3K pathways explained tumorigenesis (28) afterwards and chromosomal instability represented by 12p gain related to malignant components of NGGCTs, and poor prognosis (29). To compare, H3K27M-mutation led to multiple consequences on the chromatin landscape and DNA modification states contributed to the progression of diffuse midline glioma (DMG) (30). α-Fetoprotein (AFP) and human chorionic gonadotropin (HCG) are credible tumor markers as confirmation of yolk sac tumors and choriocarcinomas (31, 32), diagnosis and monitoring the response to treatments and follow-up, even lacking histological data (32). Nevertheless, although these two tumor indicators are fundamental in diagnosis and prediction of survival, it is challenging to make clinical decisions and arduous to foresee the outcomes for patients with normal marker levels either in peripheral blood or in cerebrospinal fluid (CSF). Increasing evidence supports that immune-related factors in the circulation have a major impact on treatment responses and clinical outcomes (33–35). Extensive immune-cell infiltration and high expression of cancer-testis antigens were commonly seen in germinoma cases. NGGCTs had significantly higher immune-cell infiltration, characterized by immune-suppression phenotype. CNS and testicular GCTs (TGCTs) both had similar mutational profiles (27). Under the circumstances, this study was designed to study lymphocyte subsets among iGCT patients and their related significance to prognosis.

The incidence of iGCT is regarded as limited with discrepancies across North America, Europe, and East Asia. The gender distribution (males=120 cases, females=25 cases, ratio: 4.8:1) in our center was consistent with the ratio of males largely exceeding females. Moreover, the cases of younger patients preceded the older in females (<16 years old: 17 patients; >16 years old: 8 patients), while the younger and the older counted for half in males (<16 years old: 60 patients; >16 patients: 60 patients). Internationally, iGCT is found to arise in the pineal (40–60%) and suprasellar (30–40%) regions or both locations as so-called bifocal (5–15%) (36). The male to female ratio is 2–3:1. In the pineal region, which is an extremely rare location in females, it is up to 15:1 (37). In our center, the incidence of common sites is about 50%, and the ratio of males to females is 4:1. Similarly, the incidence of bifocal and rare other areas was also consistent with the international incidence.

For the diagnosis of iGCTs, measurement of tumor markers is the first evaluation conducted when iGCTs was suspected. Mild-to-moderate elevations in HCG can be seen in germinoma and NGGCT, while significantly elevated HCG is a sign of choriocarcinoma. Elevated AFP is particularly seen in yolk sac tumors, while immature teratomas may show elevated HCG and AFP. If necessary, surgery is recommended during any stage of treatment to revise diagnosis. In the past, patients with presumed germ cell tumors were given 2,000 cGy of radiation to the area of abnormality or low dose of chemotherapy and if the tumor regressed after such treatment, a diagnosis of germinoma was made. Otherwise, biopsy was recommended. However, considering other tumors (eg. pineoblastomas) will also respond, the use of responsiveness to radiotherapy or chemotherapy as a diagnostic tool is now frowned upon (32).

Brain tumor patients had 3- to 8-fold lower percentages of circulating lymphocytes compared to those with melanoma or breast cancer, which is homogeneous with a recent study claiming that newly diagnosed glioma has very low numbers of T cells in peripheral blood (38, 39).Brain tumor cells rupture the blood-brain barrier, and they escape from tumor-associated antigens immune surveillance and finally lead to the entry of peripheral immune system components into the brain meanwhile communicate between tumor microenvironment and peripheral circulation (40, 41).

To our knowledge, our study is the first to report distinct lymphocyte subsets before and after chemotherapy, and first to establish an immune risk prognostic model for intracranial germ cell tumors patients. Our study confirmed that T cells and Tc cells were significantly decreased, while activated T lymphocytes collected from peripheral blood were significantly increased. It was partially contradictory with the study of D. Kempuraj, etc. They found out CD4^+^T cells and CD19^+^ B cells increased after the treatment in both benign and malignant brain tumor patients, on the contrary, CD8+T cells decreased (42). The decreasing process of CD8^+^ T cells was commonly considered called “exhaustion.” CD8+ T cell exhaustion was first reported in a study using a mouse model of chronic lymphocytic choriomeningitis virus (LCMV) infection (42), which pointed under continuously stimulation from antigens, LCMV-specific CD8^+^ T cells exhibited restricted cell proliferation and impaired immune function, compared to conventional memory CD8^+^ T cells (43). These findings have also been confirmed in human patients with cancer (44–46). Th lymphocytes bias promoted by peripheral blood exosomes and cytokines (e.g., concentrations of colony-stimulating factors 2 and 3, as well as interleukins 2, 4, and 13) was observed in peripheral blood in glioblastoma patients (47). It is consistent with the kinetics of lymphocytes in blood in our center: patients with increased Tc (CD3^+^CD8^+^) cells were inclined to present significantly encouraging survival outcomes. Furthermore, the fact that increased CD8^+^ T cells in circulation play a crucial role in anti-tumor response is confirmed in other extracranial tumors. Interestingly, NK cells did not present any significant change or relation in either course of chemotherapy or prognosis in our results. Commonly, they are responsible for cancer immune surveillance and killing by natural cytotoxicity triggered rapidly upon stimulation through germline-encoded cell surface receptors. We assumed brain tumor cells and tumor microenvironment suppressed natural killer (NK) cells *via* expression of factors such as transforming growth factor (TGF)-β and impairs NK cells by downregulating the mTOR pathway (48). Lussier DM, et al, illustrated there was no difference in the percentage of tumor-infiltrating NK cells in glioma from mice fed a ketogenic diet compared to standard diet while tumor-infiltrating NK cells produce significantly more IFN-γ and TNF (49), which indicated that alleviating immune suppression, boosting tumor-reactive immune responses or IFN-γ instead of amount of NK cells might have positive implications in treatment.

B cells (CD19^+^) induced humoral immunity in patients with brain tumors was less markedly affected. Similarly, either increasing or non-increasing B (CD19^+^) cells monitored in this study cannot indicate any outcomes. Survival analysis confirmed that increased CD3^+^T cells are related to improved survival.

Nonetheless, there are limitations of our study that should be claimed, and the results should be interpreted with prudence. Firstly, retrospective studies have limitations in nature, which cannot be adequately compensated despite usage of self-contrast and external validation of prospective study. Considering the lack of a larger sample, some of the results could not be identified as significant. We would like to expand the scale of patient participants in future research. Additionally, we did not compare the long-term dynamics of lymphocyte subsets, however it is currently in progress under surveillance and revised in another prospective program.

## Conclusion

In conclusion, lymphocyte subsets are distinct after chemotherapy in diagnosed intracranial germ cell tumors patients. Except for elevation of activated cytotoxic T cells, T lymphocytes, Tc cells, and B cells decreased after the first chemotherapy, and dynamics of Tc cells and activated T cells were closely associated with exceptional prognosis in iGCT patients and might be a potential auxiliary prognostic index (50).

## Data availability statement

The raw data supporting the conclusions of this article will be made available by the corresponding authors, without undue reservation on reasonable request.

## Ethics statement

The studies involving human participants were reviewed and approved by the Ethics Committee of Sun Yat-sen University Cancer Center (B2022-204-01). Written informed consent to participate in this study was provided by the participants’ legal guardian/next of kin.

## Author contributions

All authors contributed to the study conception and design. Material preparation, data collection and analysis were performed by HC, JC, XL, and HW. The first draft of the manuscript was written by HW. HH, ZC, YM, PC, QY, and CG critically revised the manuscript. All authors commented on previous versions of the manuscript. All authors read and approved the final manuscript.

## Funding

This research was supported by Natural Science Foundation of Guangdong province, China (2019A1515-010702) and the Science and technology Planning Project of Guangzhou (202002030114).

## Conflict of interest

The authors declare that the research was conducted in the absence of any commercial or financial relationships that could be construed as a potential conflict of interest.

## Publisher’s note

All claims expressed in this article are solely those of the authors and do not necessarily represent those of their affiliated organizations, or those of the publisher, the editors and the reviewers. Any product that may be evaluated in this article, or claim that may be made by its manufacturer, is not guaranteed or endorsed by the publisher.

## Supplementary material

Supplementary Table General characteristics of iGCTs patients in prospective study cohort. Typical sites: neurohypophysis, pineal region, and basal ganglia regions.

Supplementary Figure 1
**(A)** Mosaic plot of comparing gender, treatment patterns and evaluation of the disease. Treatment patterns were notably associated with the evaluation. **(B)** Dynamic changes of immune index of iGCTs with no significance. **(C)** Comparison of peripheral blood lymphocyte subsets in GCTs and NGGCTs before chemotherapy. **(D)** Comparison of peripheral blood lymphocyte subsets in GCTs and NGGCTs after chemotherapy. **(E)** Comparison of peripheral blood immune factors in GCTs and NGGCTs before chemotherapy. **(F)** Comparison of peripheral blood immune factors in GCTs and NGGCTs after chemotherapy.

Supplementary Figure 2
**(A)**: K-M survival analysis under gender, dynamic change of immune index and treatment in GCTs. **(C)**: K-M survival analysis under gender, dynamic change of immune index and treatment in NGGCTs. **(B)**: Forrest plot of multiple cox regression in GCTs. **(D)**: Forrest plot of multiple cox regression in NGGCTs.

## Glossary

**Table:**

iGCT	intracranial germ cell tumors
NGGCT	non-germinoma germ cell tumors
GCT	germ cell tumors
CSF	cerebrospinal fluid
AFP	α-fetoprotein
HCG	human chorionic gonadotropin
E	etoposide
P	cisplatin
B	bleomycin
C	carboplatin
I	ifosfamide
WBNT	whole-brain radiotherapy
CSI	craniospinal irradiation
GTV	gross target volume
CTV	clinical target volume
TMZ	temozolomide
OS	Overall survival
ES	embryonal stem
Th cells	T helper cells
Tc cells	T toxic
Treg cells	T regulatory cells
TNF	tumor necrosis factor
IL	Interleukin
CTL/Tc cells	cytotoxic T cells
LCMV	lymphocytic choriomeningitis virus
Bif	Bifocus of pineal gland and saddle
Mul	Multiple tumors
S-com	Single at pineal gland/basal ganglia/saddle
S-uncom	Single tumor at others sites
Ventricles	Violation of ventricle systems
Inc	Group of Increased lymphocytes
Non-Inc	Group of Non-increased lymphocytes
