# Supplementary figures and images for: Dynamic analysis of immune status in patients with intracranial germ cell tumor and establishment of an immune risk prognostic model
Source: Front Immunol. 2022 Oct 11;13:1010146. doi: 10.3389/fimmu.2022.1010146 (PMC9592720; doi:10.3389/fimmu.2022.1010146)

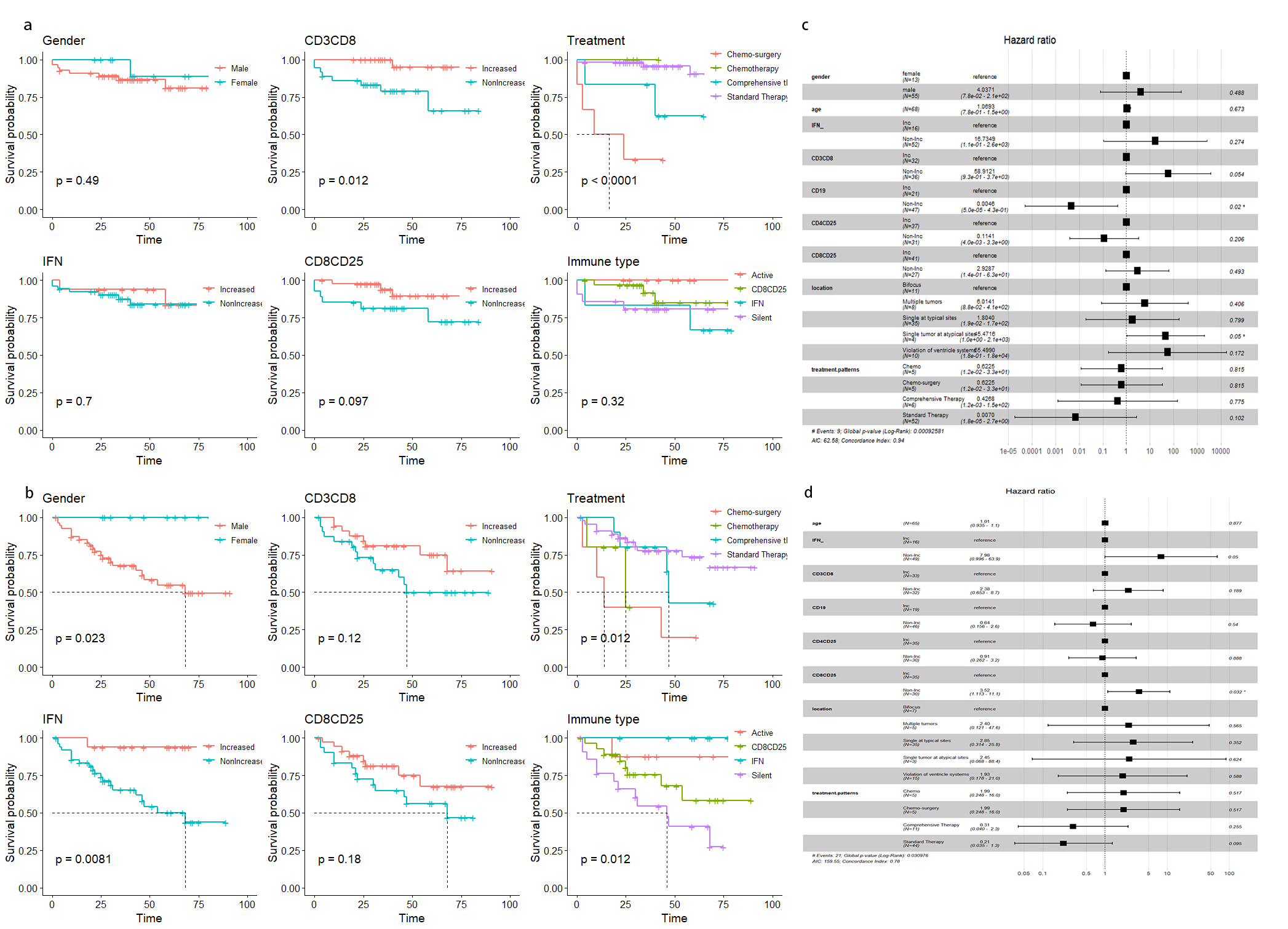

Supplement: Supplementary Figure 2 — (A): K-M survival analysis under gender, dynamic change of immune index and treatment in GCTs. (C): K-M survival analysis under gender, dynamic change of immune index and treatment in NGGCTs. (B): Forrest plot of multiple cox regression in GCTs. (D): Forrest plot of multiple cox regression in NGGCTs. [file Image_2.jpeg]
